# Environmental challenges posed by veld fires in fragile regions: The case of the Bulilima and Mangwe districts in southern Zimbabwe

**DOI:** 10.4102/jamba.v7i1.224

**Published:** 2015-11-27

**Authors:** Ernest Dube

**Affiliations:** 1 Department of Development Studies, Midlands State University, Zimbabwe

## Abstract

This original research confronted challenges to environmental management and sustainability posed by veld fires in the Bulilima and Mangwe Districts of Matabeleland in the South Province in southern Zimbabwe. Veld fires have affected the fauna and flora, polluted air and water, and destroyed livelihoods. The study aimed at establishing challenges to environmental sustainability posed by veld fires, identifying the type of environment upon which veld fires have impacted, analysing legal issues and other interventions surrounding the control of veld fires and suggesting new control measures for veld fires. A qualitative research design and quota sampling were used. The study involved 30 participants. Data was collected through a questionnaire, an interview guide and participant observation. Challenges to environmental management and sustainability posed by veld fires include property damage, reduced soil fertility, destruction of vegetation, air and water pollution and destruction of wildlife. Most veld fires are a result of human actions that emanate from the disposal of cigarettes, the burning of vegetation when preparing fields, the use of fire by hunters, smoking out bees and the making of fires by motorists along highways. The government should consider reviewing the current environmental statues. Fireguards should be wide enough to lessen veld-fire impact. Lastly, veld-fire campaigns and rehearsals should be run on a regular basis. It is hoped that this work would make a significant contribution through improving the current thinking about environmental management and sustainability, thereby benefiting policy makers, practitioners and stakeholders.

## Introduction

Veld fires pose great challenges for environmental management and sustainability in context of the development of rural communities in Zimbabwe. As such, many communities experienced massive and widespread environmental losses through veld fires. The recent increase in incidences of veld fires has been attributed to newly resettled smallholder farmers (Environmental Management Authority [EMA] 2011b). The government and stakeholders have found it difficult to manage veld fires in many parts of the country, which brought environmental sustainability into a precarious position. The protection and preservation of the environment in our societies are also emphasised in the Millennium Development Goals (MDGs), with MDG 7 meaning to ‘ensure environmental sustainability’ (Loewe 2012). Such environmental sustainability can be achieved, amongst others, through the proper management of veld fires. Bond and Mercer (2013) argue that, although veld or bushfires can rarely be prevented, effective hazard-mitigation strategies can manage and lessen the impact on humans and the environment. In the Bulilima and Mangwe Districts of the Matabeleland South Province in southern Zimbabwe, veld fires have been occurring on a regular basis. The main objective of this study was to suggest an improved approach towards environmental management and sustainability in the wake of the challenges posed by veld fires in the communal areas of the Bulilima and Mangwe districts in Zimbabwe. The sub-objectives were to:

- establish the challenges posed to environmental sustainability by veld fires
- identify the type of environment upon which veld fires have an impact
- analyse legal issues and other interventions surrounding the control of veld fires
- suggest improved control measures for veld fires to ensure environmental sustainability.

It is important, therefore, that the government and stakeholders adopt a new approach towards the control of veld fires so that the management and sustainability of the environment can be improved.

### Problem statement

Veld fires, also known as bushfires, are posing major challenges to environmental management and sustainability in the Bulilima and Mangwe Districts of the Matabeleland South Province in southern Zimbabwe. The veld fires have been taking their toll on the environment in communal areas, destroying any damageable material. Trees, species of wildlife, farming land, livestock, human lives and livelihoods suffer under the severe threat posed by veld fires. If not controlled, veld fires would result in an unclean environment, severe environmental degradation and diminished livelihoods. As such, there is need to come up with sound interventions meant to protect, preserve and sustain the environment. The Bulilima and Mangwe districts share a common boundary in Matabeleland South Province. According to the Zimbabwe National Statistical Agency (Zimstat 2012), the estimated population for the Bulilima and Mangwe districts stands at 90 501 and 66 218, respectively. Since the district is inhabited by people from the Kalanga ethnic group, they share the same tradition, culture and language. They derive their livelihood mainly from farming and from using the natural environment. Thus most of the veld fires in the Bulilima and Mangwe districts are believed to have been caused by people performing their daily activities. This has resulted in severe destruction of the veld, affecting the flora and fauna. As such, veld fires also pose some danger to human life since communities depend on the same degraded environment. Air and water pollution have been the result of such veld fires in the two districts, thereby creating health hazards that are due to the unsustainable environment. Human beings are at the centre of concerns for sustainable development, and they are entitled to a healthy and productive life in harmony with nature (United Nations 1992). It is for this reason that environmental-management activities towards a sustainable environment need to be improved in order to mitigate the effects of veld fires in the communal areas of the Bulilima and Mangwe districts.

### Literature review

In recent years, the concept of environmental management and sustainability has gained widespread recognition the world over. Efforts have been made by nations to demonstrate the serious attention that environmental management and sustainability deserve. The United Nations Conference on Environment and Development, through the Earth Summit in Rio, are testimony of governments’ commitment towards a sustainable environment. Environmental management principles set out at the first Earth Summit in Rio have been acknowledged by scientists and practitioners as well as the participating governments (Murphree 2011; World Commission on Environment and Development [WCED] 1987). The impact of veld fires thus needs to be dealt with in order to preserve and sustain the environment. Preventing veld fires may not be entirely possible, but there is a considerable scope to minimise the risk to life and property through effective management and mitigation strategies (Kiern, Franks & Verdon 2006).

Different views have been advanced by scholars regarding the concepts of environmental management and sustainability. According to Rabie (1992), there is no general agreement on exactly what the concept of environment entails. The *South African National Environmental Management Act* (NEMA), Act 107 of 1998, regards the environment as the surroundings within which humans exist and that are made up of:

i the land, water and atmosphere of the earth
ii micro-organisms, plant and animal life
iii any part or combination of (i) and (ii) and the interrelationships amongst and between them
iv the physical, chemical, aesthetic and cultural properties and conditions of the foregoing that influence human health and well-being. (n.p.)

Environmental management came about as a need to protect and preserve the environment which is under threat. This research focuses on the environment in the context of improving environmental sustainability. In theory and practice, environmental management is concerned with the protection of what is considered to be the natural and human environment (Murphree 2011). However, the ultimate goal in the implementation of environmental management is to re-balance the links between nature and humans in view of the fact that our industrial development has generated an unprecedented erosion of the environment and its natural resources (Murphree 2011). In order to understand environmental management, one has to look at what is being said about its practitioners or its characteristics (Nel & Kotzé 2009). However, environmental management has largely been viewed by Gilpin (1996), as:

a concept of care applied to individual premises, corporate enterprises, localities, regions, catchments, natural resources, areas of high conservation values, lifetime cycles, waste handling and disposal, cleaner processing and recycling systems with the purpose of protecting the environment in the broadest sense, which involves the -

- Identification of objectives
- The adoption of appropriate mitigation measures
- The protection of ecosystems
- The enhancement of quality of life for those affected, and
- *The minimisation of environmental costs*. (p.170)

It involves the identification of objectives, the adoption of appropriate mitigation measures, the protection of ecosystems, the enhancement of the quality of life for those affected and the minimisation of environmental costs (Gilpin 1995).

From the conceptualisation of environmental management above, it can be observed that effective environmental management ushers in environmental sustainability. The sustainability of the environment would mean that benefits that communities derive from the environment are lasting rather than passing. Nel and Kotzé (2009:9) posit that, for environmental sustainability to be realised, environmental management should seek to balance human demands upon the earth’s natural resource base with the environment’s ability to meet these demands on a sustainable basis. Effective environmental management contributes to sustainability as it creates healthy natural systems in which human activities are allowed to thrive sustainably (Muñoz-Erickson, Aguilar-González & Sisk 2007). Therefore, sustainable environments can only be realised through effective interventions that form part of environmental management.

Many factors have been identified as causes of veld fires in societies. These causes have resulted in veld fires of varied magnitude and effect on the natural and human environment. The natural environment has seen the destruction of the fauna and flora whilst the loss of property, pollution and at times injury have been the order of the day in the human environment. Statistics show that most of the veld fires emanated from human activities. Mkhwananzi (2007) observes that human beings have contributed to 95% of all forest and veld fires. There are only a few incidents of veld fires through natural causes (Nkomo & Sassi 2009).

The dangers that veld fires pose to the environment include the destruction of ecosystems, fauna, flora and farming land. In the context of the environment, an ecosystem is viewed as a social-ecological unit that is stable and sustainable, maintaining its characteristic composition, organisation and function over time while remaining economically viable and sustaining human communities (Costanza 1992; Rapport 1998). In rural communities, such a unit can easily be destroyed by veld fires. The EMA (2011a) observes that farming land destroyed by veld fires in Zimbabwe amounted to 950 905 hectares in 2009, 1 152 413 hectares in 2010, 713 770 hectares in 2011 and 1 320 325 hectares in 2012, thereby posing serious challenges to environmental sustainability. According to Chagutah (2011), land is central to the rural development and environmental challenges facing the southern-African region. By destroying land, veld fires are a threat to food security in southern Africa since land is one of the most vital assets for millions of poor people in the region (Clover & Eriksen 2009). Veld fires also result in the decline of veld conditions and an increase in air pollution, thereby reducing the quality of air that people breathe (Dube 2015; Everson, George & Schulze 1989).

Apart from affecting the natural environment, veld fires also impact on the human environment. Household assets, including livestock and capital assets, are destroyed by veld fires. This hazard is also a threat to human life as it may result in death or injury. Veld fires deprive people of a clean environment by causing pollution. They pollute the environment in which people live, posing a serious health hazard through ash that can be blown everywhere. It therefore becomes a mammoth task in rural communities to maintain a sustainable environment in the wake of the challenges posed by veld fires.

Various controls in environmental management and sustainability have been practiced globally to tackle veld fires in a bid to maintain a sustainable environment. Such interventions include a stakeholder approach and the use of fireguards. The stakeholder approach entails that stakeholders are drawn from different government departments, local authorities and affected communities. The stakeholder theory includes environmental stewardship since the environment can be affected by human or corporate activity (Ohreen 2010). The advantage of this theory is its balancing of the interests of various groups, including the affected communities, for mutual gain. However, the theory is fraught with challenges since balancing stakeholders’ interest may be difficult. It is also a challenge to determine stakeholders with legitimate interests, given the many groups and individuals involved.

### Research method and design Setting

The research was done in the Bulilima and Mangwe districts of Matabeleland South in Zimbabwe. The two districts have a combined estimated population of 156 719 (Zimstat 2012).

### Design

Qualitative research was the methodology chosen to inform this study. According to Kvale (1996), qualitative research is regarded as progressive research. He adds that this type of methodological approach is sensitive to human situations and involves emphatic dialogue with studied subjects. The study employed an interpretivist paradigm. The approach of interpretivism involves the study of phenomena in their natural settings, and it also regards respondents as experts (Unlin, Robinson & Tolley 2005).

### Sampling

Participants were chosen through quota sampling. This sampling method entails choosing two strata in the form of members of the community and government officials from the studied population. In this case, participants were chosen from the two districts of Bulilima and Mangwe. From each stratum, a quota was drawn so that each group was represented. This method was ideal for the study as it improved the representation of particular groups within the population and ensured that the strata were not over-represented. A total of 30 participants were involved in the study. These consisted of members of the community, officials from the EMA, police officers and staff from the Fire and Ambulance Service. Both men and women were chosen since they experience the impact of veld fires equally. Twenty participants who are members of the community were chosen from the two districts (10 from each district) to give information on the effects of veld fires on the environment. These are the people directly affecting and being affected by environmental degradation through veld fires. The same sampling method was applied to choose officials with a duty to protect the environment. This quota consisted of 10 participants chosen from departments that deal with veld fires. Such departments included EMA, the Zimbabwe Republic Police (ZRP) and the Fire and Ambulance Services. Officials from EMA and officers from ZRP were chosen for their expertise and experience in enforcing environmental legislation whilst staff from the Fire and Ambulance Services were chosen for their expertise in managing veld fires.

### Procedure

A qualitative research design uses mainly questionnaires, interviews and observations to explore and understand the attitudes, opinions, feelings and behaviour of individuals or groups (Du Plooy 2001; Henning 2003). In this study, questionnaires, interviews and observations were adopted as methods of primary-data collection. Questionnaires were chosen because they can be completed by respondents in their own spare time. Leedy (1989:142) argues that questionnaires offer the researcher the ability to ‘… observe data beyond the physical reach of the observer’. They are also generally less expensive to administer since they involve mere distribution and have a low drain on time and finance. Questionnaires with open-ended questions were distributed to officials from EMA, police officers and fire tenders so that they could give their input on the law-enforcement mechanisms relating to the environment and interventions used in controlling veld fires. The use of open-ended questions was meant to solicit divergent views from the participants. Questionnaires and open-ended questions were also an ideal instrument for this group since most government officials face little challenges in completing them as they possess high levels of literacy.

An interview is a two-person conversation initiated by the interviewer for the purpose of obtaining research data focused on content specified by the research objectives, namely systematic description, prediction or explanation (Cohen, Manion & Morrison 2000:24). An interview guide was used to investigate the experiences, views and opinions of members of the community affected by veld fires. In this case, the guide helped address the objectives by seeking to establish the challenges to environmental sustainability posed by veld fires and to identify the type of environment upon which veld fires have an impact. This data-collection instrument was chosen as it saves time through merely asking questions from participants and writing down their responses. Most members of the community are not comfortable with other data-collection methods like questionnaires because of their low level of literacy, hence an interview guide was ideal. Interviews allow researchers to produce a rich and varied set of data through a thorough examination of experiences, feelings and opinions (Kitchin & Tate 2001). Community members easily understood questions in the interview guide and provided different thoughts about the effects of veld fires on the environment in the Bulilima and Mangwe districts. From their narrations, the researcher was able to deduce their feelings concerning experiences of past incidents of veld fires.

The researcher also visited places that were affected by veld fires to make on-site observations. An observation guide was used, and it detailed the main things to be observed. These included the type of environment affected by veld fires and the livelihoods involved. The guide also needed the researcher to make observations concerning the physical control measures used by the community in mitigating the impact of veld fires. Every detail of what was observed was written down by the researcher. This method has the advantage of bringing the researcher’s own experience to the study. The data gathered from participant observation enhances the study’s credibility.

## Results

Results from data obtained from the field are presented in this section. The section highlights the challenges posed by veld fires to environmental sustainability, identifies the type of environment upon which the veld fires had an impact and analyses legal issues surrounding control of veld fires. Veld fires pose serious challenges to the environment and sustainability in the Bulilima and Mangwe communal areas. Data collected from respondents are presented in the form of tables and figures.

### The challenges that veld fires pose to environmental sustainability

The destruction of the environment has been the major challenge posed by veld fires in the Bulilima and Mangwe districts. Veld fires in the Bulilima and Mangwe districts have affected the environment negatively through destruction and pollution (Table 1).

Twenty members of the community were interviewed in order to highlight, from their lived experiences, the challenges caused by veld fires. They were asked to indicate one major factor which they thought had a negative effect on the environment in the Bulilima and Mangwe districts. Out of 20 respondents, 4 (20%) felt that veld fires have led to property damage, 2 (10%) indicated that soil fertility has been affected, 5 (25%) said that the fires destroy vegetation, 2 (10%) indicated that they cause air pollution, 2 (10%) said that they pollute water sources, and 5 (25%) said that veld fires destroy wildlife (Table 1). These findings agree with results of studies by the World Wide Fund (WWF) (2001) and Nyamadzawo et al. (2013) who found that the outbreak of veld fires have resulted in increased loss of agricultural produce, a reduced availability of food for both humans and animals, a reduced growth rate of vegetation and loss of equipment.

**TABLE 1:** Challenges to sustainability caused by veld fires.

Challenges posed by veld fires	Frequency (out of 20)	Percentage
Damage to property	4	20
Soil fertility	2	10
Destruction of vegetation	5	25
Air pollution	2	10
Water pollution	2	10
Wildlife	5	25
Total	20	100

**FIGURE 1:**
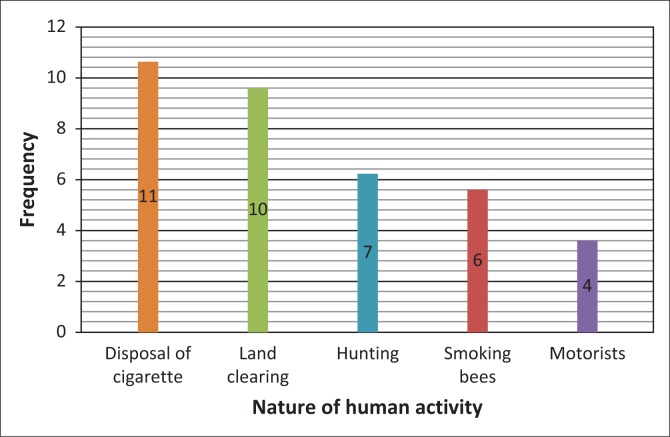
Human activities causing veld fires.

Having indicated the major impact of veld fires, respondents went on to reveal what they thought were the major causes of veld fires in their communities. Most veld fires in the Bulilima and Mangwe districts are a result of actions by human beings (Figure 1).

As can be observed (Figure 1), most veld fires occur through careless human activities that include burning of vegetation during field preparation for the coming farming season, improper disposal of cigarette stubs, hunting, smoking out bees and motorists making fires along highways. Improper disposal of cigarette stubs and the burning of vegetation during land preparation are major causes of veld fires. Of the 20 respondents, 11 indicated the careless disposal of cigarette stubs as a major cause of veld fires, 10 indicated burning of vegetation for land preparation as cause, seven thought that hunting for wild animals was the cause, six indicated smoking out bees whilst four indicated that some fires are caused by motorists along roads. These findings resonate well with a study by Nkomo and Sassi (2009:11) in which they found that ‘… most of the veld fires are man-made and only a paltry 1% has been due to natural causes such as lightning’. A closer look at these statistics reveals that improper disposal of cigarette stubs and burning of vegetation during field preparation are the two major causes of veld fires (Figure 1).

### The type of environment most impacted by veld fires

Respondents who work in organisations or departments with a mandate to deal with veld fires were given self-administered questionnaires with open-ended questions. From the questionnaires given, respondents showed that there are two types of environment upon which veld fires have the biggest impact – the natural and human environments.

Six (60%) of ten respondents indicated that veld fires have the biggest impact on the natural environment whilst four (40%) indicated that it is the human environment that suffers the biggest impact. The natural environment includes all types of vegetation, all animal species and their surroundings. The human environment consists of places where people live and where they are likely to be found, their property and everything that they have built. However, respondents were of the opinion that both types of environment are heavily affected even though respondents had disparate views on the intensity of the impact on each.

**FIGURE 2:**
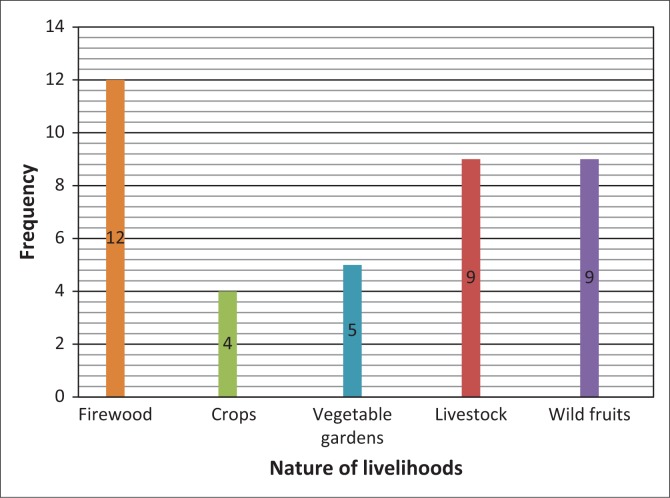
Nature of livelihoods affected by veld fires.

In the two types of environment, various livelihoods are seen to be affected by veld fires. These include crops, vegetable gardens, livestock, firewood and wild fruit. Data on the nature of livelihoods most affected by veld fires were obtained from the members of the community as shown (Figure 2).

As can be seen in Figure 2, 12 of the 20 respondents felt that firewood is the most affected livelihood. Nine indicated wild fruit, and another nine indicated that livestock was the most affected. Crops (four respondents) and vegetable gardens (five respondents) were perceived by respondents to be the least affected by veld fires in the Bulilima and Mangwe districts.

### Legal issues and other control measures surrounding veld-fire management

Through the literature review and the data collected from government officials, it was observed that there are statutes that govern uncontrolled veld fires in Zimbabwe. Such statutes include the *Environmental Management Act*, Chapter 20:27; the *Rural District Councils Act*, Chapter 29:13; the *Parks and Wildlife Act*, Chapter 20:14; and the *Forestry Act*, Chapter 19:09. Part IX of the *Environmental Management Act* deals with standards of environmental quality (Government of Zimbabwe 2002a). According to the *Environmental Management Act*, Chapter 20:27, no person is allowed to light a fire outside of residential or commercial premises during the period 31 July to 31 October of each year (Government of Zimbabwe 2002a). The *Rural District Councils Act*, Chapter 29:13, bestows custodianship of the local natural environment or of resources upon the Rural District Councils (Government of Zimbabwe 2002b). The Act also empowers the RDCs to enact by-laws with regards to such issues as amenities and facilities, water and its pollution, effluent and solid waste management and the removal of vegetation (Government of Zimbabwe 2002b). The *Forestry Act*, Chapter 19:09, creates a commission for the administration, control and management of state forests. According to Mudekwe (2007), this Act also prohibits smoking in a state or private forest, and anyone who throws away burning material shall be guilty of an offence and is liable to a prescribed fine or 6 months in jail or both. From field observations, it was noted that fireguards are put in place as an intervention or control measure to veld fires were not wide enough and not adequate to mitigate the impact of veld fires. According to the *Forestry Act*, a fireguard is deemed not sufficient unless it stretches for 9 m on either side of a fence (Government of Zimbabwe 2002c). From their experience in environmental law enforcement, government officials were of the opinion that current legislation is not deterrent enough because of the high number of human activities causing veld fires.

Apart from legislation, the interviews revealed other veld-fire control measures that have been used in the Bulilima and Mangwe districts to manage veld fires. The frequency of these practices was indicated by stakeholder respondents (Table 2).

**TABLE 2:** Commonly practised veld-fire control measures.

Challenges posed by veld fires	Frequency (out of 20)	Percentage
Damage to property	4	20
Soil fertility	2	10
Destruction of vegetation	5	25
Air pollution	2	10
Water pollution	2	10
Wildlife	5	25
Total	20	100

*N* = 10.

The ten respondents gave their thoughts on the importance of control measures as follows: Four respondents (40%) preferred fireguards, two respondents (20%) preferred campaigns, two respondents (20%) preferred law enforcement, one respondent (10%) preferred public education, and one respondent (10%) preferred rehearsals (Table 2). Fireguards, campaigns and law enforcement are the most common veld-fire interventions in Bulilima and Mangwe whilst public education and rehearsals are rare (Table 2). These results explain why veld fires continue to pose challenges to environmental management and sustainability in the two districts. These findings agree with results from previous studies. The National Fire Protection Association (2000) concurs that fireguards are a common intervention practised to limit the spread of veld fires. Public education and rehearsals are not common in the two districts. These should be adopted and implemented for containing veld fires and for environmental management. Greater environmental awareness and a stronger sense of responsibility lead to sustainable development (ADEA 2010). Results from previous studies by Mitchell et al. (2013) also show that relevant educational content can help to provide the knowledge and skills needed for making informed decisions. A renewed and improved approach by the government and stakeholders is strongly needed to ensure environmental sustainability.

## Ethical considerations

Research ethics were observed in carrying out the study. This had the aim of improving participation by the respondents. A university clearance, permitting the researcher to conduct the study, was obtained and made available to all respondents.

The potential benefits from the study are available for the government and its stakeholders. They would benefit from the new approach towards environmental management and sustainability. This study entailed no risks or harm that could have affected the participants. All participants were assured that no physical or psychological harm would emanate from the study. They were also assured that no hazards were associated with data collected from them.

The recruitment procedure for the study was based on voluntarism with none of the participants forced to provide information. The participants were advised that they were entitled to withdraw their consent at any stage of the study if they so wished to do so.

Full consent was obtained from participants before the study was conducted. Their consent was checked and verified before they participated to make sure that it was real. This was to make sure that participants had no reservations and that their consent was not unduly influenced.

Data obtained from the respondents in both Bulilima and Mangwe were treated with the utmost confidentiality. Data were carefully handled so that there was no unauthorised access, and such data were used solely for the purposes of the study.

### Trustworthiness

To ensure the trustworthiness of the data collected, the study first obtained information from members of the community since they are the ones affected by veld fires. These provided first-hand information from their lived experiences, namely being affected by the phenomenon. Officials from EMA and police officers being the law enforcers also provided data from their experiences in enforcing legislation related to environment conservation. These are experts in the field of law enforcement. The trustworthiness of the data was further enhanced by visiting places affected by veld fires in order to make on-site observations.

### Practical implications

This study provides news ways of dealing with veld fires, thereby helping governments and stakeholders in improving environmental sustainability. The suggested interventions include the review of the current statues for veld fires, the creation of fireguards conforming to the widths laid down in the statutes and regular campaigns. These interventions have the potential to inform policy and practice.

### Limitations to the study

Limitations to this study included resistance from some respondents, who seemed unwilling to release information, as well as the large size of the study area to be covered. To ensure that the reliability of the results was not affected, the purpose of the research was first explained to the participants in order to increase their response. A high degree of confidentiality was also assured for the participants. For easy coverage of the study area, the researcher stayed within the communities.

### Recommendations

Having analysed and considered the findings, a new approach towards veld-fire management and environmental sustainability should not be taken for granted. The study, therefore, recommends that the government should consider a review of the current statues for veld fires so that the statutes become stricter, thus deterring more would-be offenders. Fireguards should be intensified, and these should also conform to the widths laid down in the statutes of the country. More campaigns and rehearsals should be undertaken in order to educate the communities about the dangers of veld fires and equip stakeholders with the necessary expertise to deal with veld fires. This view sits well with the thinking of Forsyth, Kruger, and Le Maitre (2010), namely that the ability of a community to manage a veld fire should be considered. Future research should focus on improving environmental sustainability through the indigenous knowledge systems of local communities.

## Conclusion

Veld fires are posing serious challenges to the environment and sustainability in the communal areas of the Bulilima and Mangwe districts of Matabeleland South in Zimbabwe. It was the respondents’ feelings that veld fires destroy crops, soil fertility, vegetation and animal species. According to the participants to the study, most of the veld fires in the said communal areas emanate from careless human activities. Respondents’ opinions were that such activities include the burning of vegetation in field preparation, the use of fire by hunters, smoking out bees, improper disposal of cigarette stubs and the making of fires by motorists along highways. From their experiences, the burning of vegetation in field preparation and the improper disposal of cigarette stubs are the major causes of veld fires in the Bulilima and Mangwe communal areas. Members of the community were of the view that veld fires impact negatively on both the natural and human environments. However, some respondents thought that their impact on the natural environment is more severe. The livelihoods of communities such as crops, vegetable gardens, livestock, firewood and wild fruit are destroyed by ravaging veld fires as perceived by the study participants. The government of Zimbabwe put in place supporting legislation to regulate conduct that could cause veld fires. Such legislation includes the *Environmental Management Act*, Chapter 20:27; the *Rural District Councils Act*, Chapter 29:13; and the *Forestry Act*, Chapter 19:09. According to government officials responsible for law enforcement who participated in the study, these measures have not been effective and are not able to deter enough people. Respondents revealed other veld-fire control measures that have been used, which include fireguards, campaigns, public education and rehearsals. Respondents felt that these too have not been effective. A new approach towards veld-fire management and environmental sustainability is recommended. The government should consider a review of the current legislation so that is becomes more deterrent to would-be offenders. Fireguards should be properly used, and fireguards that conform to the width gazetted in the statutes should be adopted. Regular campaigns should be run in the communities. Lastly, rehearsals also need to be run to equip stakeholders with the necessary skills.
